# Bearingless Inertial Rotational Stage for Atomic Force Microscopy

**DOI:** 10.3390/mi15070903

**Published:** 2024-07-11

**Authors:** Eva Osuna, Aitor Zambudio, Pablo Ares, Cristina Gómez-Navarro, Julio Gómez-Herrero

**Affiliations:** 1 Departamento de Física de la Materia Condensada, Universidad Autónoma de Madrid, 28049 Madrid, Spain; eva.osuna@uam.es (E.O.); aitor.zambudio@uam.es (A.Z.); cristina.gomez@uam.es (C.G.-N.); 2 Condensed Matter Physics Center (IFIMAC), Universidad Autónoma de Madrid, 28049 Madrid, Spain

**Keywords:** piezoelectric inertial motion, rotational stage, nanotechnology, AFM, positioning

## Abstract

We introduce a novel rotational stage based on inertial motion, designed to be lightweight, compact, and fully compatible with atomic force microscopy (AFM) systems. Our characterization of this stage demonstrates high angular precision, achieving a maximum rotational speed of 0.083 rad/s and a minimum angular step of 11.8 μrad. The stage exhibits reliable performance, maintaining continuous operation for extended periods. When tested within an AFM setup, the stage deliveres excellent results, confirming its efficacy for scanning probe microscopy studies.

## 1. Introduction

One of the first devices that allowed macroscopic displacements based on piezoelectrics was developed by C. Gerber, G. Binnig, and H. Rohrer in the early 1980s [1].

The goal was to provide a precise tip-sample approach system for Scanning Tunneling Microscopy. This device, called the “louse”, consisted of a triangular piezoelectric element. Each vertex contained a small metallic disk that acted as a capacitor plate. The movement was achieved through a sequence of piezoelectric stretches followed by the application of a high voltage to the metallic plates, which fixed the previous or subsequent capacitors accordingly.

A few years later, Dieter Pohl designed a device based on inertial movement [2], which has since served as the foundation for numerous piezoelectric-based devices to produce macroscopic displacements with sub-nanometric precision [3] (and references therein).

The operating principle of all these devices can be illustrated with the following macroscopic example: Consider a coin placed on a hardcover book. Hold the book and observe the position of the coin relative to the body. Next, slowly extend the arm; the coin, due to friction with the book cover, remains in the same position relative to the book but moves away from the body. When the arm reaches full extension, quickly retract it to the initial position. If the acceleration caused by this second movement is large enough, the coin will slip relative to the book cover and end the extension/contraction cycle farther than its initial position. Repeating these stick/slip cycles will cause the coin to move further away until it eventually falls off the book. If the arm is replaced with a piezoelectric element and the coin with the object to be moved, an inertial displacement system based on piezoelectrics is obtained. The movement is performed by applying a periodic voltage signal to the piezoelectric element, oscillating between a minimum voltage (V_min_) and a maximum voltage (V_max_). Initially, the voltage changes from V_min_ to V_max_ slowly and smoothly (stick phase). Upon reaching V_max_, the signal drops abruptly within a very short time (typically a few microseconds) to the minimum voltage (slip phase). The simplest example of such a waveform is a sawtooth signal. This approach, combined with bearings, has been also used to fabricate rotatory stages [4]. In the following sections, we will describe the design and construction of a bearingless rotary stage based on “shear piezoelectrics”. Our approach simplifies previous designs and guarantees a constant friction force between the rotor and the mechanical force transducer.

## 2. Motivation: In-Situ Atomic Force Microscopy (AFM) Rotation and Twisted Two-Dimensional (2D) Stacking

AFM is a versatile tool (WSxM v5.0 Develop 10.3 software) for measuring the various physical properties of materials with nanoscale resolution [5], and is particularly useful for atomically thin 2D materials. AFM operates in several modes, including contact, amplitude and phase modulation, intermittent contact, electrical, electrostatic, magnetic, capacitive, and many others [6].

**Figure 1:**
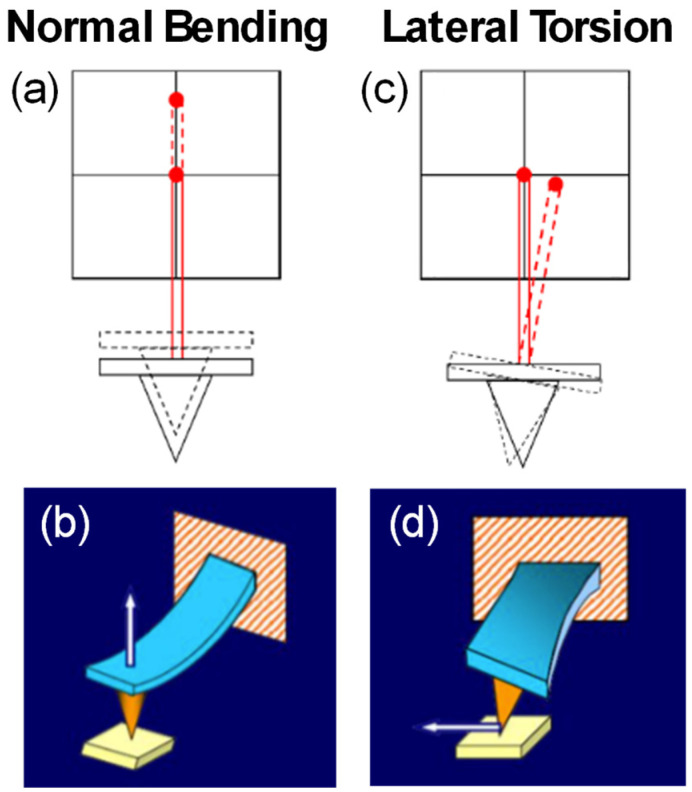
Schemes depicting general AFM operation. (**a**,**b**) Cantilever and laser beam deflection induced by the tip-sample normal force, showing the variation in the four-quadrant photosensor detection. (**c**,**d**) In LFM mode, the friction force between the tip and sample during scanning produces a lateral twist in the cantilever, known as the lateral force (LF) signal. From this LF signal, the friction force between the tip and sample can be inferred.

In contact mode, the force sensor undergoes two main deflection modes: normal bending (Figure 1a,b), which is the usual control signal for topography imaging, and lateral bending (Figure 1c,d), which provides friction images. Lateral force microscopy (LFM) is the AFM operation mode that quantifies the friction force between the tip and the sample by measuring the lateral torsion of the cantilever produced when the tip slips over the sample surface while in physical contact [7]. The LFM technique allows for the quantification of local friction with atomic resolution.

It is quite common for the friction properties of a material to depend on the sample orientation, and the correct analysis of the friction can be delicate [8]. As seen in Figure 1d, the lateral torsion of the cantilever occurs along the scanning direction. Although the majority of AFM setups allow for variation in the scanning angle, the relative alignment between the cantilever axis and scanning direction is particularly critical in LFM experiments. When the scanning direction is not perpendicular to the long axis of the cantilever, the sensitivity and reproducibility of the LFM method are adversely affected. In the worst-case scenario, when the tip scans parallel to the long axis of the cantilever, there is no lateral contribution to the cantilever bending, and the detected LF signal vanishes. Hence, studying the friction in a particular sample direction requires aligning this direction with the AFM scanning direction. The implication is that the sample must be rotated at will, calling for a compact, stable, and precise rotational stage compatible with the stringent requirements of high-resolution AFM (see Supplementary Figure S1 for additional details).

Another significant application of such a rotary system lies in the field of 2D materials stacking. In recent years, there has been a growing interest in studying the properties of twisted 2D atomic layers, leading to numerous remarkable results [9,10,11]. This trend has given rise to new fields, such as twistronics, and related techniques. To achieve sample rotation with sub-degree precision during the stacking process, a small, easy-to-handle rotary system with such accuracy could enable the easy, fast, and replicable preparation of twisted moiré materials. This advancement would advance the field and pave the way for scalable applications.

## 3. Device Description

**Figure 2:**
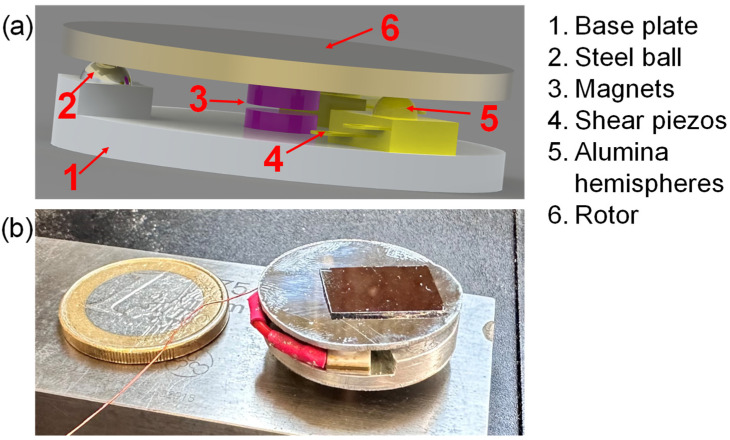
(**a**) A 3D render showing the design and elements of the rotational stage. (**b**) Picture of the real device with an AFM sample set on the rotor. A EUR 1 coin is shown for size comparison.

Our device consists of two discs, each 25 mm in diameter, referred to as the base plate and the rotor (Figure 2). The thickness of these discs is 2 and 1 mm, respectively, although the base plate can vary between 1 and 3 mm.

On the base, we attached a pair of 5 × 5 × 1.8 mm^3^ “shear piezos” (Thorlabs PL5FBP3, Thorlabs, Inc., Newton, NJ, USA), and on each of them, an alumina hemisphere 2.5 mm in diameter (Thorlabs KDESP, Thorlabs, Inc., Newton, NJ, USA). These two hemispheres and a metal ball made of steel are distributed at equal angles of 120 degrees. The shear piezos are lined up to produce an in-phase tangential motion. The steel ball provides electrical contact between the base plate and the rotor. As we ground the base, the rotor is also grounded through the steel ball, ensuring that there are no electrostatic issues in the AFM imaging. Additionally, we fixed cylindrical magnets (in purple in Figure 2), 4 mm in diameter and 2 mm in height, to the center of both discs. When the discs are stacked, the north and south poles of the magnets are aligned and separated by a 0.2–0.3 mm air gap. Consequently, the rotor rests on the ball and the two hemispheres, forming a plane, while the magnets create a strong attraction between the discs, increasing the frictional force between the rotor and the balls and providing a well-defined rotation axis.

We observed that the material for the base is not critical, so we used aluminum. For the rotor, a hard material is preferred to avoid wear. Initially, we also used aluminum, but the rotation was irregular. Subsequently, we tested X50CrMoV15 stainless steel (Heritage Steel, Clarksville, TN, USA) and discs of old computer hard drives (aluminum with a hardened layer to protect the magnetic information). In both cases, the results were excellent, and we were able to keep the rotor spinning for many hours without apparent degradation. Therefore, we conclude that the friction between the balls and the rotor is not an issue once the rotor surface is hard enough to withstand wear.

The total mass of the device depends on the thickness and materials used but ranges between 7.4 and 3.5 g and the height ranges between 8 and 4.5 mm. We used epoxy adhesive (Araldite Rapid, Hunstsman Corporation, The Woodlands, TX, USA) to glue the piezos, the magnets, the ball and the alumina hemispheres.

## 4. Device Characterization

We conducted a series of experiments to characterize the performance of the inertial rotational stage and assess its suitability for AFM. Specifically, we examined the angular velocity and step angular displacement as functions of the driving frequency.

The driving voltage was consistently set at 300 V. Considering the calibration of the shear piezo, this should have resulted in a tangential motion of about 1 µm. The driving frequency was varied between 10 Hz and 7 kHz. We employed a conventional function generator to produce the sawtooth wave, along with an NF Corp HSA4051 (NF Corporation, Yokohama, Japan) high-speed bipolar amplifier capable of delivering a maximum current exceeding 1 A. Since piezoelectrics can be modeled as capacitors, achieving the microsecond timescale for rotor slip events necessitates both a high voltage and high current.

**Figure 3:**
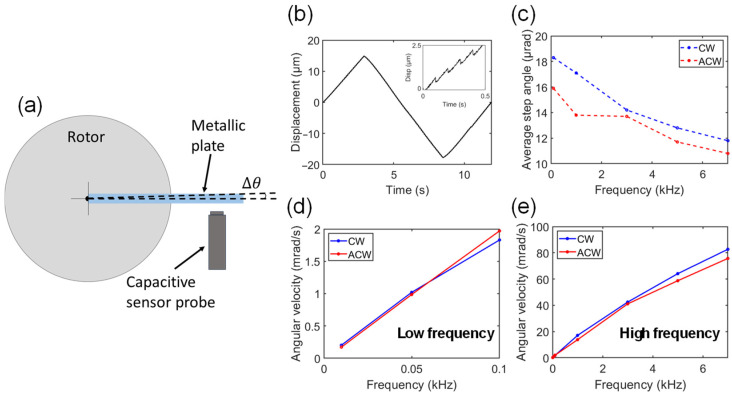
(**a**) Capacitive displacement sensor setup used to measure angular displacement. (**b**) Linear displacement at 10 Hz, clockwise and counterclockwise. Inset showing the stepping behavior of the piezoelectric actuators. (**c**) Angular step angle vs. frequency of the rotor in the clockwise (CW, blue) and counterclockwise (ACW, red) directions. Angular velocities at low (**d**) and high (**e**) frequencies.

We conducted characterization experiments at low frequencies (10–100 Hz) using a capacitive displacement sensor (capaNCDT 6200 controller with CS02 probe, Micro-Epsilon, Ortenburg, Germany), as illustrated in Figure 3a. The capacitive displacement sensor, which operates on the principle of a parallel plate capacitor, is limited to measuring linear displacements on metallic surfaces. To facilitate the measurement of angular displacement, we attached a lightweight grounded metallic plate to the rotor. The rotation angle was then calculated based on the displacement of this plate, given that the plate’s displacement (in microns) is minimal compared to the 30 mm distance between the capacitive sensor and the rotor’s center. Figure 3b displays the linear displacement measured by the capacitive sensor when the piezoelectric actuators were operated at 10 Hz, first in the counterclockwise direction, then clockwise, and then counterclockwise again.

From these experiments, we found that the angular velocity exhibits a nearly linear dependence on the driving frequency at both low and high frequencies (see Figure 3d,e). Although there were slight differences between the clockwise (CW) and counterclockwise (ACW) directions, the overall trend remained consistent in both. The maximum angular velocities were 83 mrad/s (CW) and 76 mrad/s (ACW), both observed at 7 kHz. This maximum velocity translates to nearly 1 rpm. However, for AFM purposes, a continuous rotation speed was not a relevant parameter since the maximum angle to be rotated in AFM is ±180°. Instead, the device’s mechanical stability and its ability to rotate a sample to a specific angle with precision are crucial. The system’s compactness and micro-control of rotation are also particularly advantageous for AFM applications.

A more relevant parameter for AFM is the step angle of the piezoelectric actuator. We observed that the step angle decreased slightly when the frequency was increased due to slippage (see Figure 3c). The maximum step angle of ~18 µrad occurred at 10 Hz (CW), while the minimum step angle of 11.8 µrad was obtained at 7 kHz (ACW). This resolution is more than sufficient for most AFM applications, and for much more demanding uses where rotational adjustments are required, such as for magic-angle graphene, which involves angle precision in the order of 0.1° [9,12] (0.00192 rad), which is two orders of magnitude above our minimum step size.

Video S1 in the Supplementary Materials shows the device operation in real time. Video S2 shows the stability and reliability of the rotational stage along 3 h (the speed of the video has been increased at a factor of 256).

## 5. Application in AFM Experiments

**Figure 4:**
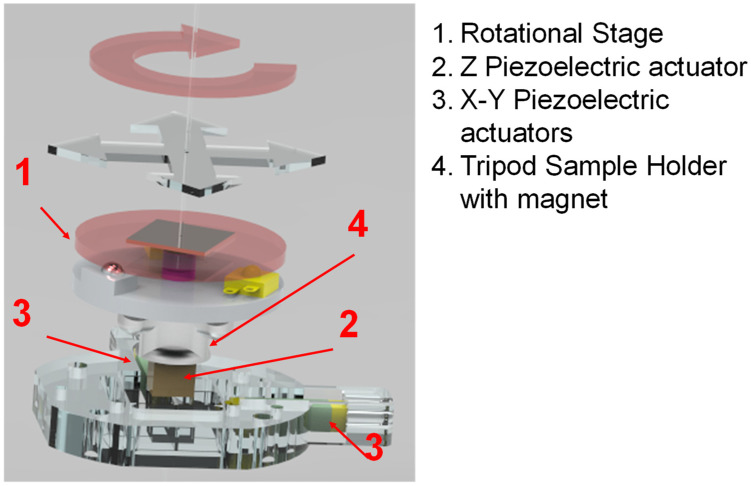
Render image showing the AFM scanner and the rotational stage, highlighting the sample motion. For the sake of clarity, we have added labels to each component.

In addition to the macroscopic characterization, we tested the performance of our setup in real AFM experiments. To achieve this, we placed the device on top of an AFM scanner (Figure 4).

This scanner consists of an aluminum frame that houses two piezoelectric actuators, each measuring 11 × 3 × 3 mm^3^, which move the sample in the xy plane. A third piezoelectric actuator, measuring 5 × 5 × 5 mm^3^, moves the sample in the z-direction, perpendicular to the sample plane. On top of this z piezoelectric actuator, we have attached the usual AFM sample holder consisting of a light tripod with a magnet in the middle and three stainless steel balls. In order to set the rotational stage on this holder, we glued a magnetic stainless steel plate (25 mm diameter 0.6 mm thick) to the base plate to be attracted by the magnet. By applying sawtooth waves to the xy piezoelectric actuators, we can move the rotator inertially within the sample plane. This allows us to rotate and translate the sample placed on top of the rotor (represented by the arrows in Figure 4). Since the area of interest is never exactly at the axis, the ability to inertially position the sample within the plane is crucial.

**Figure 5:**
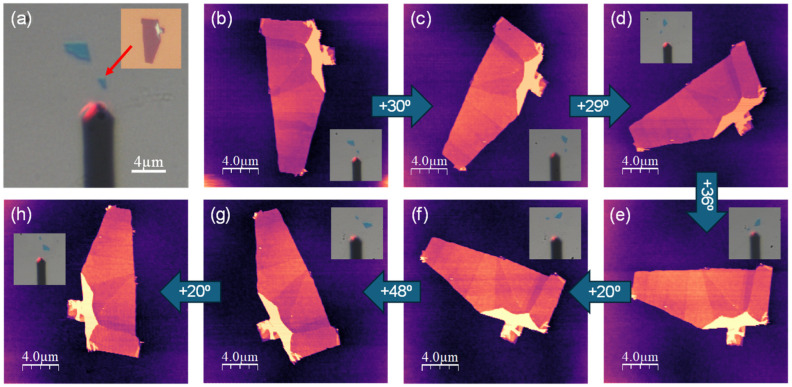
(**a**) Optical microscope image of the two few-layer graphene flakes’ starting position with respect to the AFM cantilever. Inset: higher-resolution optical image of the flake under study. (**b**–**h**) Topographic AFM images of the rotation steps, from the zero degrees initial flake position in (**b**) to the 180° twisted image in (**h**). The magnitude of each rotation step is provided inside the blue arrows. Insets: optical images where the rotation of the flake with respect to the fixed cantilever can be observed.

To test and validate the performance of the rotational stage for AFM imaging, we measured a few-layer graphene flake deposited on a SiO_2_/Si substrate. Figure 5 outlines the step-like 180° rotation of this sample. This configuration, combined with the use of an optical microscope for the cantilever sample visualization, allows us to easily rotate and reposition the sample multiple times.

**Figure 6:**
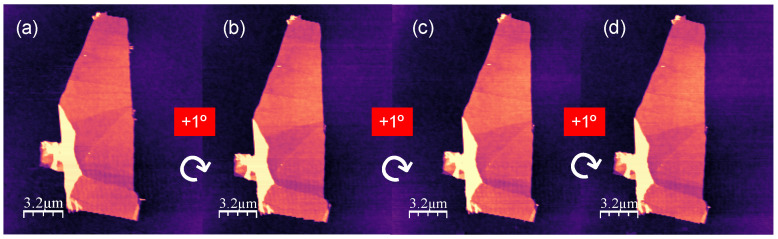
(**a**) AFM topographic image showing the same flake as in Figure 5 in its initial position. (**b**–**d**) Same flake as in (**a**) after consecutive 1° clockwise rotation steps.

We can select the rotation angle in various ways: by using optical references if there are large flakes around the substrate, or by calibrating the rotational stage with fine-tuned parameters for controlled steps. The feasibility of this latter method is shown in Figure 6.

To test the accuracy of our device within the AFM setup, we systematically rotated our sample in 1-degree steps using appropriate input parameters, as depicted in Figure 6. Although this is a proof-of-concept result, it demonstrates that we can reduce the rotation step to the micro-degree level, enabling precise control of the sample’s rotation angle. This configuration and methodology offer significant flexibility and accuracy in manipulating the fine-tuned parameters, and in performing controlled steps.

It is important to highlight that the stability of our AFM was not compromised in any way by the use of the rotation device, as evidenced by the stability of the images. Additionally, we did not observe any increase in thermal drift or a reduction in the image acquisition frequency.

**Figure 7:**
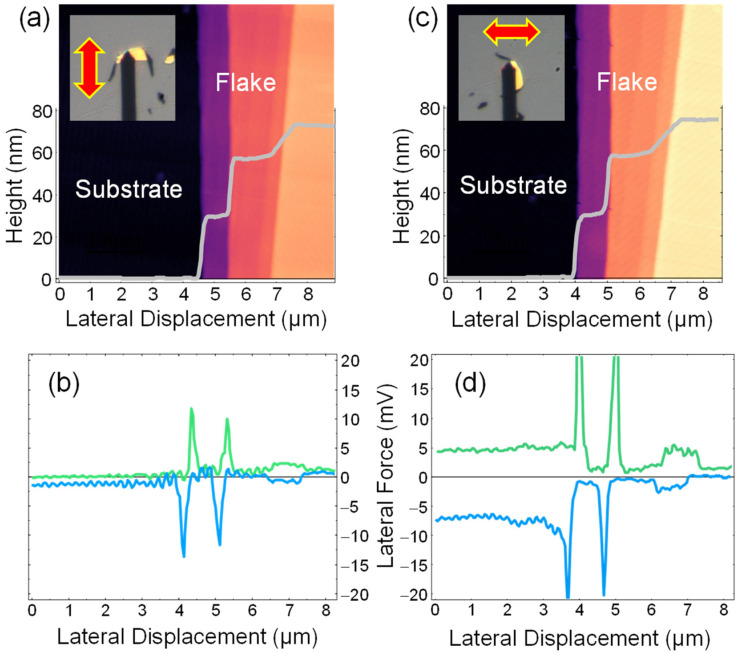
(**a**,**c**) AFM topography images acquired in contact mode, including the average height profiles (gray curves), depicting the substrate (black region) and the edge of a graphite flake with visible terraces. The insets illustrate the relative position between the cantilever and the flake, with red arrows indicating the fast-scan direction. The AFM image in (**a**) has been rotated 90° to facilitate comparison with the AFM image in (**c**). (**b**,**d**) The corresponding average friction loops for the images above. Green and blue lines correspond to forward and backward scan along the fast scan direction, respectively.

Finally, we evaluated the suitability of the rotational stage for acquiring friction images. Figure 7 presents a selected graphite flake, with contact AFM topography images acquired with the sample edge positioned perpendicular (Figure 7a) and parallel (Figure 7c) to the long axis of the cantilever. In both cases, the fast-scan direction is indicated by the red arrows in the insets. As anticipated, the topography images are nearly identical. However, the corresponding friction loops (Figure 7b,d) display a significant difference. Specifically, Figure 7d shows an increased friction signal, resulting in a larger friction loop and a pronounced distinction between the substrate and graphite friction. Although Figure 7b exhibits noticeable peaks, these are attributed to a second-order effect, as explained by Salmeron et al. [8].

## 6. Conclusions

We have designed, built and tested a compact inertial rotational stage for AFM applications. The stage exhibits high reliability and high precision, as measured by optical microscopy and capacitive sensing. The stage is very stable and allows AFM imaging without compromising performance. Further improvements will include an encoder to independently measure the angular position and miniaturization. In addition, the stage is an optimum solution for other applications such as the fabrication of moiré materials.

## 7. Methods

AFM details: we use WSxM software (WSxM v5.0 Develop 10.3 version) for both controlling the AFM and processing the acquired data [13,14]. Our system consists of a homemade AFM working in air conditions and controlled by a Dulcinea Electronic unit from Nanotec Electronica S.L., Tres Cantos, Spain. The AFM images in Figure 5 and Figure 6 were acquired in dynamic mode working with amplitude modulation (AM-AFM), and the images and friction profiles in Figure 7 were acquired in contact mode. All data were acquired using a Point-Probe-Plus–FMR AFM tip from Nanosensors, Neuchatel, Switzerland (nominal spring constant 2.8 N/m, and resonance frequency 75 kHz). 

Sample preparation: Graphene was deposited by mechanical cleavage and exfoliation from natural macroscopic graphite (flaggy-flakes, NGS Naturgraphit GmBH, Leinburg, Germany) on a silicon substrate with a thermal grown silicon oxide layer of 300nm thickness. We inspected the samples using an optical microscope, namely Zeiss Axio Imager.A2m (Carl Zeiss AG, Oberkochen, Germany).

## Data Availability

The data that support the findings of this study are available upon request from the corresponding authors.

## Supplementary Materials

The following supporting information can be downloaded at: https://www.mdpi.com/article/10.3390/mi15070903/s1, Figure S1: schematics of a cantilever scanning a grid along perpendicular directions. Video S1: device operation at real speed (piezo driving frequency = 7 kHz); Video S2: device operation at ×256 showing continuous operation for 3 h.
