# Supplementary figures and images for: Bearingless Inertial Rotational Stage for Atomic Force Microscopy
Source: Micromachines (Basel). 2024 Jul 11;15(7):903. doi: 10.3390/mi15070903 (PMC11279377; doi:10.3390/mi15070903)

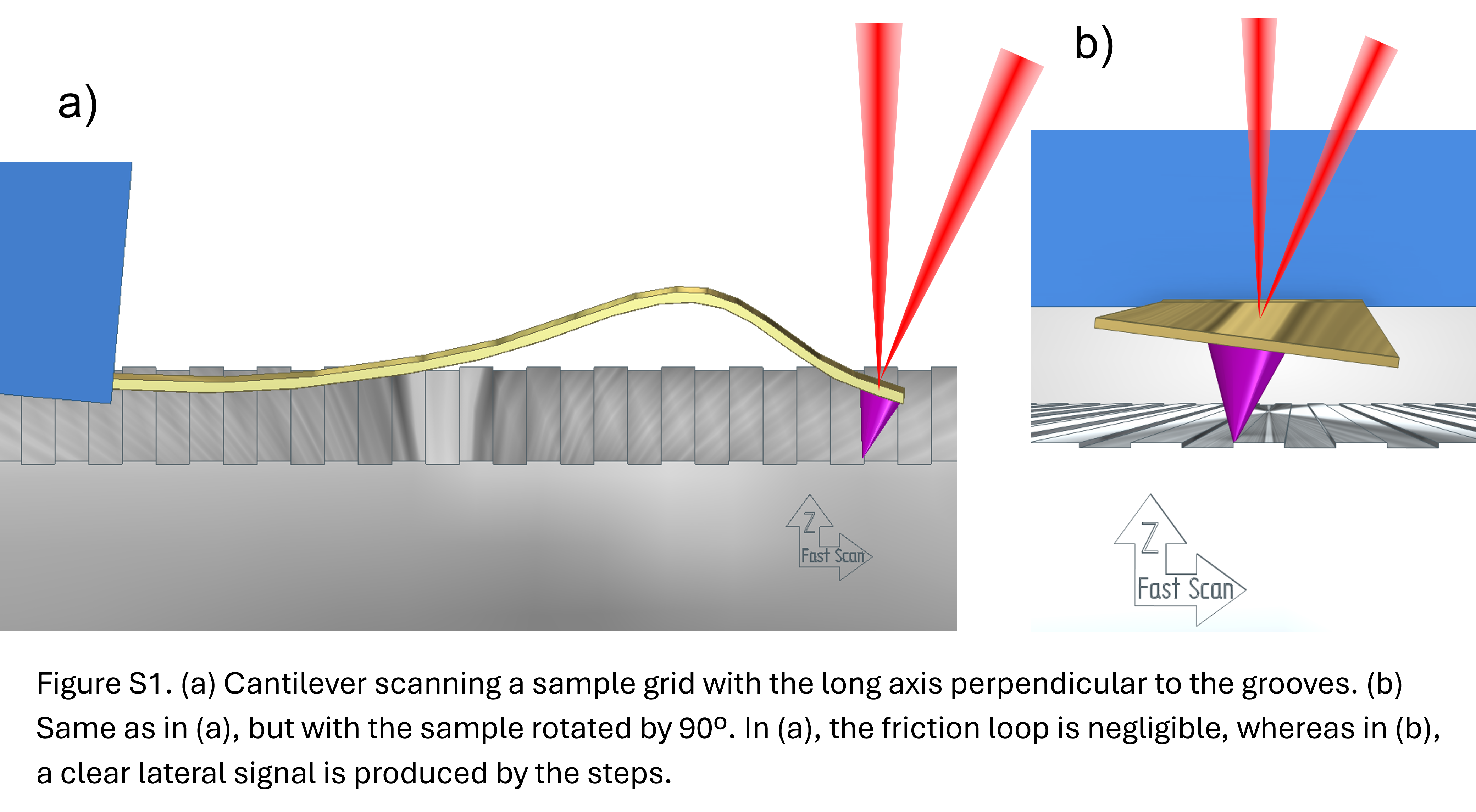

Supplement: Supplementary file 1 [file micromachines-15-00903-s001.zip › FigureS1.png]
